# Simultaneous Bilateral Testicular Metastases from Renal Clear Cell Carcinoma: A Rare Presentation in Von Hippel–Lindau disease

**DOI:** 10.15586/jkcvhl.v9i2.211

**Published:** 2022-05-05

**Authors:** Asaad Moradi, Delaram Farhoumand, Behnaz Bouzari, Behnam Shakiba

**Affiliations:** 1 Department of Urology, Firoozgar Hospital, School of Medicine, Iran University of Medical Sciences, Tehran, Iran;; 2 Department of Pathology, School of Medicine, Iran University of Medical Sciences, Tehran, Iran

**Keywords:** neoplasm metastasis, renal cell carcinoma, von hippel lindau disease, testis

## Abstract

In this article, we present a Von Hippel–Lindau (VHL) patient with synchoronus bilateral testicular metastasis from renal cell carcinoma (RCC). A 50 year-old man, a known case of VHL syndrome was referred with palpable masses in both the testes. His medical history demonstrated that he had undergone the brain surgery for cerebellar hemangioblastoma. He had undergone simultaneous Whipple’s pancreatectomy and left radical nephrectomy becuase of well-differentiated neuroendocrine tumors in head and body of the pancreas and a 6-cm clear cell-type grade-3 RCC in the left kidney. Scrotal sonography demonstrated vascular and heteroechogen masses measuring 19×14 mm in lower pole of the right testicle, 19×16 mm in upper pole of the right testicle, and 23×16.5 mm in upper pole of the left testicle. After having patient’s consent, bilateral orchiectomy was performed by inguinal incision. Histopathologic examination and immunohistochemistry staining revealed metastasis from RCC. The most common neoplasm of reproductive system in VHL patients is epididymal papillary cystadenoma. Owing to it’s benign nature, the management is conservative with routine physical examination and ultrasonography. Our patient indicated that every scrotal mass in patients with VHL is not to be considered as epididymal papillary cystadenoma.

## Introduction

Von Hippel–Lindau (VHL) is an autosomal-dominant neoplastic syndrome. It is caused by genetic aberration of tumor suppressor gene *VHL*. It is characterized by multi-organ neoplastic lesions such as hemangioblastoma in central nervous system (CNS), renal cell carcinoma (RCC), and neuroendocrine tumors. By the age of 60 years, up to 70% of patients develop RCC (1). Most common sites for RCC metastasis are the lungs, lymph nodes, bones, and liver, although testicular metastasis from RCC is rare and bilateral testicular metastasis is extremely rare (2,3). In this article, we present a VHL patient with synchoronus bilateral testicular metastasis from RCC.

## Case Presentation

A 50-year-old Iranian man, a known case of VHL syndrome, was referred to urology clinic with palpable masses in both the testes. Physical examination of both the testes revealed atrophy, and two masses in the right testis and one in the left testis were palpable.The masses were firm to hard, and fixed to the underlying structures with less than 2 cm in diameter. No other abnormalities were noted. His medical history revealed that he had undergone surgery of the brain for cerebellar hemangioblastoma. He had undergone simultaneous Whipple’s pancreatectomy and left radical nephrectomy because of well-differentiated neuroendocrine tumors in head and body of the pancreas and a 6-cm clear cell-type grade-3 RCC in the left kidney. Renal tumor was confined to the kidney (stage T1N0M0).

**Figure 1:**
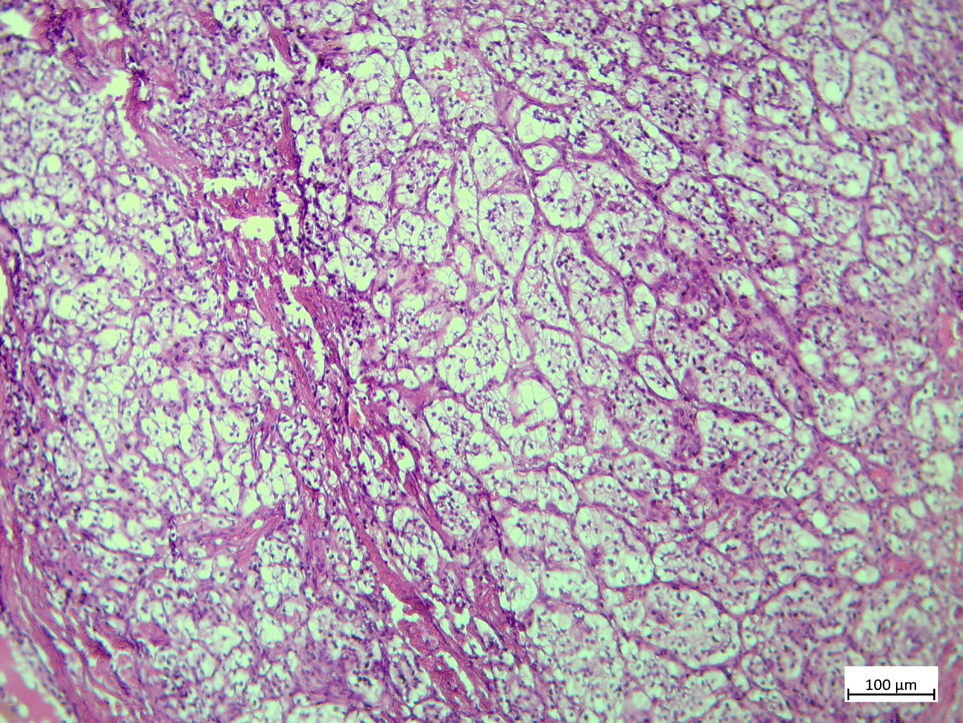
Histopathological examination and immunohistochemistry staining revealed bilateral testicular metastases from renal clear cell carcinoma.

Laboratory tests, including hormonal examination, tumor markers, and semen analysis, were performed. Luteinizing hormone (LH) and follicle-stimulating hormone (FSH) were increased and serum level of testosterone was significantly low. Tumor markers, including lactate dehydrogenase (LDH), alpha-fetoprotein (AFP), and Beta human chorionic gonadotropin (B HCG) were within normal range. Semen analysis revealed azoospermia.Abdominal ultrasonography revealed no abnormal finding. Scrotal sonography demonstrated vascular and heteroechogen masses measuring 19×14 in lower pole of the right testicle, 19×16 mm in upper pole of the right testicle, and 23×16.5 mm in upper pole of the left testicle. After having patient’s informed consent, bilateral orchiectomy was performed by inguinal incision. Histopathologic examination and immunohistochemistry staining revealed metastasis from RCC (Figure 1). Patient was referred for systemic therapy with sunitinib malate (Sutent).

## Discussion

Von Hippel–Lnidau syndrome is a autosomal-dominant neoplastic syndrome. It is induced by mutation of VHL tumor suppressor gene located on the short arm of chromosome 3. VHL patients develop multi-organ neoplastic lesions such as CNS hemangioblastoma, pancreatic neuroendocrine tumors or cysts, RCC or cysts, retinal hemangioblastoma, epididymal cystadenomas, pheochromocytoma, and endolymphatic sac tumors. Clear cell renal carcinoma manifestations appear in 70% of VHL patients up to 60 years of age (1). Common sites of RCC metastasis are the lymph nodes, lungs, bones, liver, and brain. However, metastasis to the testes is rare (2).

Generally, secondary neoplasm of the testes is uncommon. Owing to lower temperature of the scrotum than other parts of the body and presence of blood–testis barrier, which is formed by sertoli cell to protect spermatozoas, scrotum environment is not appropriate for development of metastasis (4).

Only 39 cases of testicular metastasis have been reported since 1946. Most of these metastasis involved ipsilateral and the left testis. Bilateral testis metastasis of RCC is extremely rare, especially synchoronous testicular involvement. Based on our knowledge, only two patients have been reported; The first case was reported by Moriyama et al. (2) in 2013 and the second one by Wang et aI. in 2020 (3). Both cases were VHL patients. Our patient was the third case of VHL syndrome with bilateral testis metastasis of RCC.

The most common neoplasm of the reproductive system in VHL patients is epididymal papillary cystadenoma (5). Differential diagnosis of cystadenoma includes cystadeno carcinoma, epididymal cysts, and intratesticular varicocele. This cystadenoma is a asymptomatic solid tumor with slow growth (6). Owing to benign nature of cystadenoma, the management was conservative with routine physical examination and ultrasonography (1,5). Our patient and two previous patients revealed that every scrotal mass in patients with VHL is not considered as epididymal papillary cystadenoma. More evaluation is required for early detection and treatment of probable metastasis. If metastasis and malignancy are not ruled out definitely, inguinal radical orchiechtomy and histopathological assessment are recommended.
